# The Expression of EPOR in Renal Cortex during Postnatal Development

**DOI:** 10.1371/journal.pone.0041993

**Published:** 2012-07-26

**Authors:** Lu Xiao, Zhanyong Li, Pengjuan Xu, Zhigui Li, Jing Xu, Zhuo Yang

**Affiliations:** 1 College of Medicine, Nankai University, Tianjin, China; 2 College of Life Sciences, Nankai University, Tianjin, China; Imperial College London, United Kingdom

## Abstract

Erythropoietin (EPO), known for its role in erythroid differentiation, has been shown to be an important growth factor for brain and heart. EPO is synthesized by fibroblast-like cells in the renal cortex. Prompted by this anatomical relationship and its significant impact on the maturation process of brain and heart, we asked whether EPO could play a role during the development of renal cortex. The relationship between the development of renal cortex and the change of EPO receptor (EPOR), through which EPO could act as a renotropic cytokine, became interesting to us. In this study, the day of birth was recorded as postnatal day 0(P0). P7, P14, P21, P28, P35, P42 and mature mice (postnatal days>56) were used as the animal model of different developmental stages. Immunohistochemistry and Western blotting were used to detect the expression of EPOR in mouse renal cortex. Results showed that expression of EPOR decreased with the development of renal cortex and became stable when kidney became mature. The expression of EPOR was detected at the renal tubule of all developmental stages and a relatively higher expression was observed at P14. However, at the renal corpuscle the expression was only observed at P7 and quickly became undetectable after that. All these suggested that a translocation of EPOR from renal corpuscle to renal tubule may take place during the developmental process of renal cortex. Also, EPO may be an essential element for the maturation of renal cortex, and the requirement for EPO was changed during postnatal development process.

## Introduction

Erythropoietin (EPO), a hormone-like substance that can promote the generation of red blood cells, is mainly secreted by the fibroblast-like cells in the renal cortex. It is initially highlighted for its indispensable action on the hematopoietic system. The major function of EPO is mediated by the specific cell-surface receptor, erythropoietin receptor (EPOR). Targeted disruption of EPO-EPOR system caused utero death in mice between embryonic days E11 and E13, because of lacking definitive erythropoiesis in the fetal liver [1], [2]. These suggested that the vital role of EPO–EPOR signaling was in the proliferation, survival and terminal differentiation of erythroid progenitors, and it might also play as an important developmental factor. Findings of EPOR expression indicated a role of EPO in non-haematopoietic tissues such as the brain [3], [4] retina [5], kidney [6], smooth muscle cells [7], myoblasts [8], vascular endothelium [9] and heart [10]. Ubiquitous distribution of EPOR in non-erythroid cells was associated with the diverse biological functions for EPO in non-haematopoietic tissues [11]. Recent years, more and more additional nonerythropoietic tissue/organ developmental properties of EPO have become the focus of research. EPOR signaling is required for normal brain development. EPO acts directly to stimulate neural progenitor cells and to prevent apoptosis in the embryonic brain [12]. Also the increased apoptosis was observed in the myocardium of EPOR-null mouse during the embryonic development [13]. All these suggested that EPO could not only be regarded as a hematopoiesis-related cytokines, but also might play a significant role during the development process of non-haematopoietic tissues.

As we all know, kidney is closely associated with EPO, especially after birth when the site of haematopoiesis switches to the bone marrow, the kidney becomes the predominant EPO producing organ [14], [15], [16]. Recently, recombinant EPO forms (epoetinalfa, epoetin-beta and the long-acting analogue darbepoetinalfa) have been widely used for treatment of anaemia in chronic kidney diseases. At the same time, a novel renoprotective action of EPO has been proposed [17] [18], [19], [20]. Cultured kidney cells can be protected by EPO against ischemic acute renal injury. It can be an *in vitro* evidence for EPO' protective action [20]and also can be a direct link between EPO and kidney.

EPO could play as an important growth factor in many specific non-hematopoietic organs. Simultaneously, EPO is protective for kidney cortex, and kidney is the place secreted EPO. However, at present most studies have only concentrated on the protective effect of EPO barely on its developmental influence on kidney cortex. The relationship between the development of kidney cortex and the change of EPOR through which EPO could take effect on became interesting to us. Previous studies have shown that human and mouse kidney cells express functional erythropoietin receptors [6]. In this study, we focused on the changes of EPOR expression at different postnatal developmental stages in mouse kidney cortex using immunohistochemistry and Western blotting methods.

## Materials and Methods

### 1. Ethics statement

The animal experiments were performed in accordance with institutional guidelines, and the study was approved by the ethics committee of Nankai University. Experiments were designed to minimize the number of animals used and their suffering.

### 2. Antibodies and reagents

Rabbit polyclonal anti-EPOR antibodies (primary antibody, Santa Cruz Biotechnology, Inc. CA, U.S.A); Alexa 488 conjugated goat anti-rabbit IgG antibodies (secondary antibody, Invitrogen, San Diego, CA, U.S.A); rabbit polyclonal anti-β-actin IgG (primary antibody, Santa Cruz Biotechnology, Inc. CA, U.S.A); chemiluminescent HRP substrate (Immobilon Western, Millipore Corporation, Billerica, MA, U.S.A).

### 3. Animals

All animal procedures were accorded to the protocols approved by the Animal Care Committee of the Animal Center at the Chinese Academy of Sciences in Shanghai. Adult kunming mice (8–10 weeks old) were purchased from the Experimental Animal Center of the Chinese Academy Medical Sciences. Animals were maintained under standard laboratory conditions under artificial 12 hours light/12 hours dark cycle. Two females were paired with one male (2∶1) for a period of 4–5 days until mating. The day of birth was recorded as postnatal day 0 (P0). P7, P14, P21, P28, P35, P42 and mature mice (postnatal days>56) were used as animal models of different postnatal developmental stages. In order to avoid the influence of the red cells and other cells in the blood, we took measures of perfusion before collecting materials. Kidneys were collected at different time points.

### 4. Immunofluorescence staining

Samples were fixed by 4% paraformaldehyde in phosphate buffer (PBS,pH 7.4) at 4°C over 4 hours, immersed in 30% sucrose overnight at 4°C and then embedded in OCT compound (Tissue-Tek, Miles). Cryosections (5 µm thickness) of kidney were cut (Leica CM 1850, Leica Instruments) and mounted on gelatin-coated slides for immunostaining. Sections were washed three times with PBS 5 minutes for each and incubated with 10% normal goat serum with 0.3% Triton X-100 in PBS for 1 hour at room temperature. Then Incubations with appropriate primary anti-EPOR (working dilution 1∶500) were performed overnight at 4°C. After repeated washing with PBS three times for 10 minutes each, sections were incubated with Alexa 488-conjugated (working dilution 1∶1000) anti-rabbit IgG for 1 h at 37°C, washed with PBS three times for 10 minutes each, coverslipped, and examined under the fluorescence microscope. For analyze of EPOR expression, fluorescent intensity was quantified by measuring intensity in the tissue using Image-Pro Plus. Data were analyzed from three sections of one sample, and there were six samples for each age group.

### 5. Western blotting

The kidneys were isolated from bodies, and the cortex was isolated by the dissection, and then minced into pieces and lysed in lysis buffer containing a proteinase inhibitor cocktail (1∶100 dilutions). Lysates were centrifugated at 12000 r/min for 10 minutes at 4°C. The supernatant was collected and total proteins were quantified by bicinchonic acid assay according to manufacturer's instructions. Semiquantitative immunoblotting was carried out as followed: Proteins were separated on polyacrylamide gels by SDS-PAGE and transferred to polyvinylidene difluoride (nitrocellulose) membrane in Tris-glycine buffer at 300 mA for 1 hour. Membranes were blocked with 5% fat-free milk blocking buffer for 2 hours at room temperature and then incubated overnight at 4°C with the respective primary rabbit polyclonal anti-EPOR antibodies (working dilution 1∶1000) and rabbit polyclonal anti-β-actin IgG (working dilution 1∶2000). After that, membranes were washed three times with Tris-buffered saline/Tween 20 (TBST) buffer and exposed to horseradish peroxidase-conjugated secondary antibodies (working dilution 1∶2500) for 1 hour at the room temperature. Blots were washed another three times with TBST and detected with chemiluminescent HRP substrate (Immobilon Western). Figures showed representative results from experiments repeated at least three times.

### 6. Statistics

All data were expressed as mean ± standard error mean (SEM) and analyzed by SPSS 17.0. There was a minimum of six animals per age group. Results were analyzed by one-way ANOVA and P<0.05 was considered significant.

## Results

### 1. Immunofluorescence detection of EPOR in the mouse renal cortex

**Figure 1:**
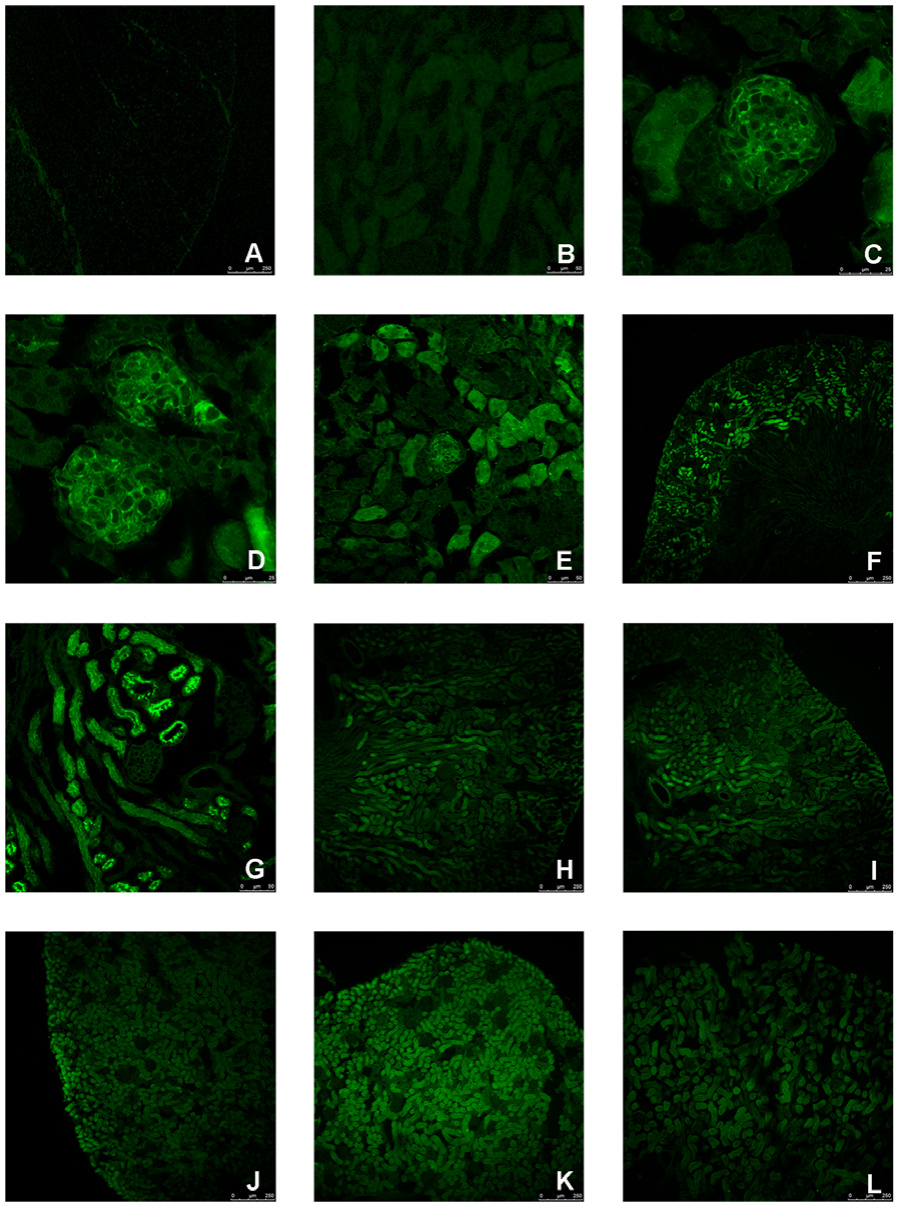
Localization of EPOR during mouse kidney cortex postnatal development. The negative control was shown in (A, B). The expression of EPOR in the mouse kidney cortex could be detected at P7 (C). Suspected Immature corpuscle could be observed at P7 (D). The expression of EPOR was higher at tubule part, and the expression at corpuscle part was almost undetectable at P14 (G). The expression of EPOR was detected in the kidney cortex at P7 (E), P14 (F), P21 (H), P28 (I), P35 (J), P42 (K) and mature mouse (L).

**Figure 2:**
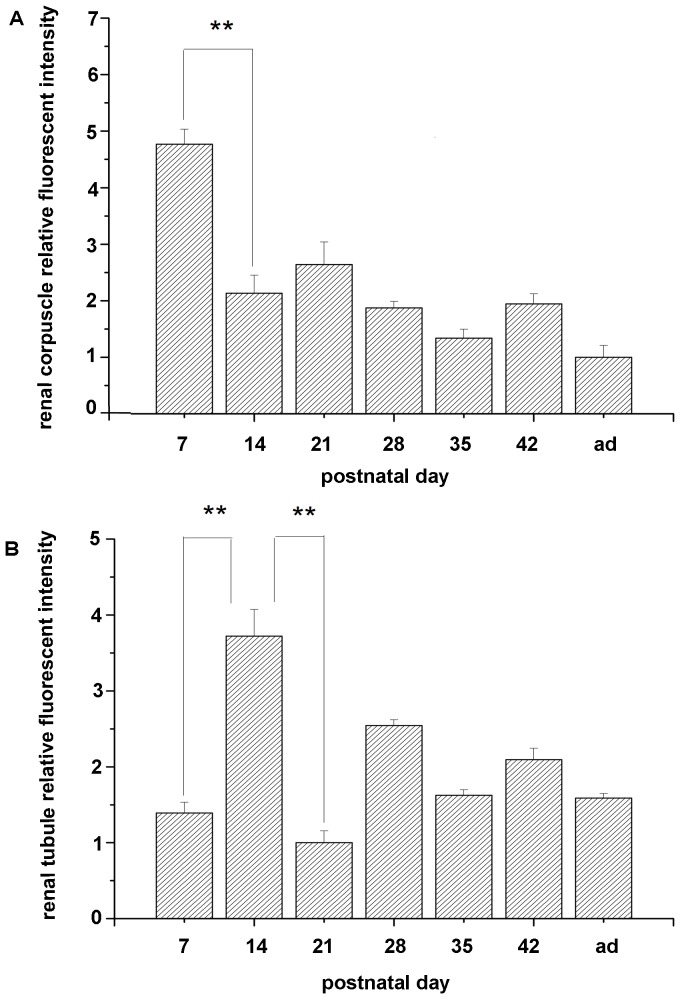
Relative fluorescent intensity of renal corpuscle part (A) and renal tubule part (B).

To identify the morphological and developmental changes in protein expression, the specific antibody was used to mark the EPOR detection during the postnatal development in mice. EPOR can be detected in the renal corpuscles at P7 (Figure 1. C), and at the same developmental stage, suspected immature glomerulus could be observed (Figure 1. D). It is known that there are four developmental stages of renal corpuscles: comma-shaped body, S-shaped body, renal corpuscles of stage III and renal corpuscles of stage IV [21]. At P7, there were mostly renal corpuscles of stage IV, but some immature renal corpuscles were observed (Figure 1. D). At P14, there was a relatively higher expression in the renal tubule (Figure 1. G) and the expression in glomerulus was nearly undetectable (Figure 1. G). After P14, the expression in renal corpuscle can hardly be observed and the relatively higher expression at renal tubule disappeared. In addition, the expression of EPOR became relatively stable after P35. Moreover, the volume of cortex was increased after P7. In summary, the expression of EPOR at renal corpuscle decreased rapidly during the postnatal development (Figure 2. A) and the expression of EPOR at renal tubule increased at first stage, and dropped dramatically after P14 (Figure 2. B).

### 2. Western blotting detection of EPOR in the mouse renal cortex

**Figure 3:**
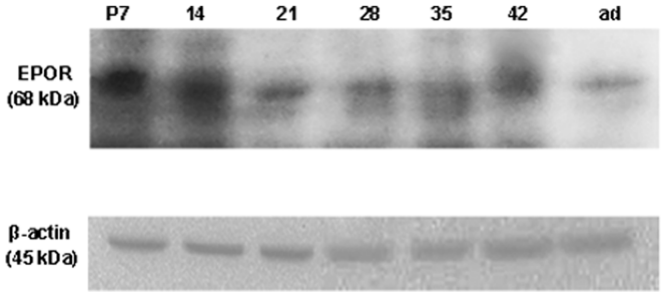
Immunoblot analysis of EPOR in mouse kidney cortex during the development. The position of 68 kDa molecular size was the expression pattern of EPOR in renal cortex at the indicated ages. β-actin was used as a consult. When the expression of EPOR was normalized to β-actin, the level was found decreased with development.

To identify the developmental changes in protein expression at renal cortex, the expression of EPOR was examined by Western blotting as β-actin was a consult. For this experiment, the cortex was separated from the kidney. As seen in Figure 2, immunoreactive bands of 68 kDa were regarded as the expression of EPOR. It was found that the expression of EPOR was decreased. Meantime, during this period, the peak expression was at P7. The expression level was nearly the same between P7 and P14. Meanwhile, there was a drop between P28 and P35 and it was relatively stable after P35 (Figure 3).

## Discussion

In the present study, immunohistochemistry and Western blotting were used to detect the expression of EPOR in the renal cortex of P7, P14, P21, P28, P35, P42 and mature mice. Results showed that: (1) the expression of EPOR was decreased during the postnatal development. The peak expression of EPOR was at P7 and the expression level was almost the same between P7 and P14, and it was relatively stable after P35. (2) EPOR was expressed in the renal corpuscle at P7 and quickly became undetectable after that time. Meantime, there was a relatively higher expression of EPOR in the renal tubule than that of glomerulus at P14, and then it dropped dramatically after P14. All these results indicated that there was a translocation of EPOR during the postnatal development and the expression was decreased, and became relatively stable after P28.

EPO, a hormone necessary for production of red blood cells, function primarily to transport oxygen, is synthesised by the adult kidney cortex, and the production can be induced by anaemia or hypoxic stress [22]. It has been proved that EPO is an essential cytokine for the brain [12] and heart [10] development. At the same time, EPO exists in several EPOR-expressing nonhemopoietic cells, which shows anti- apoptotic, mitogenic and differentiation-inducing effects [23], [24]. In brain, production of erythropoietin has been detected in astrocytes and neurons [25], [26]. Also it has been reported that EPO shares structural homology with growth hormone [27]. As a result, more and more studies have focused on its developmental effect. In EPOR-null mice, increased apoptosis was seen in the fetal brain as early as embryonic day E10.5 [12]. In heart, EPOR was first detected during midgestation [10] and persisted through adulthood [28]. Mice with targeted deletion of EPO or EPOR exhibited apoptosis in endocardium and myocardium, and a reduction in the number of proliferating cardiomyocytes at embryonic day E12–E13 [13]. Observations of *in vivo* development suggested that endothelial cells of the kidney originated from external blood vessels which grew into the developing kidney [29]. It has been proved that EPO has a direct impact on the blood vessels [9]. According to these facts, EPO might also act on the kidney. In this study, we have concentrated on its developmental influence on the renal cortex. The dramatically fall of the EPOR expression in renal cortex has attracted our attention. It has been demonstrated that EPOR expression in the brain is decreased quickly during the development [3], consistent with the variation tendency we have discovered in the renal cortex. Bacallao R *et al* has demonstrated that the vast majority of differentiated mature renal cells in the healthy kidney is maintained in the ‘quiescent state’ and can therefore not be readily stimulated to enter the cell cycle [30]. Therefore, we suspected that EPO was not required to work as a proliferation regulation factor when the cells became mature. It is possibly reasoned for the low expression of EPOR after P35 when most of renal cells become mature and lost the ability of proliferation. This finding agreed with Liu *et al*'s result that functional erythropoietin receptor was undetectable in mature renal cells [3].

There are two other results that have aroused our interest. Firstly, the rapidly fall of EPOR expression in the renal corpuscles after P7. Secondly, the higher expression of EPOR at renal tubule at P14. There are four developmental stages of renal corpuscles: comma-shaped body, S-shaped body, renal corpuscles of stage III and renal corpuscles of stage IV [21]. Some researches indicated that the number of the cells at corpuscles was proliferated prominently from P1 to P7. After P7, the developing characteristic of renal corpuscles was marked by volume growth of average unit. As a result, P7 can be considered as an important time point of the development of renal corpuscles, after that most of the corpuscles stop proliferating. All these can indicate why the expression fell rapidly after P7 as the corpuscle developmental process almost stopped and most of the glomerulus becomes mature, so EPO could suspend its developmental influence on glomerulus cells. When it comes to the renal tubule cells, it has been reported that they don't stop actively proliferating until P14 [21], [31]. As a result, P14 can be regarded as a turning point of the tubule development. Therefore, EPOR, through which EPO could take effect, decreased in the renal tubule after P14. Thereafter, tubule cells slow down but don't stop its proliferation process. It can explain the continuous EPOR expression at all developmental stages of mouse kidney tubule. But why it has a higher expression at P14 remains unknown.

We noticed that the expression of EPOR could be observed at every stage of development and there was a dramatically fall during the development. Meantime, a translocation of EPOR can also be detected during development. In conclusion, the expression of EPOR through which EPO could take effect is in keeping with the proliferation process of either corpuscle or tubule. So we surmised that EPO in the kidney cortex may act as an important proliferation regulation factor. All these findings may add a novel annotation of EPOR for the function during the postnatal development of kidney cortex, which provided a new handhold for us to understand the development of kidney cortex and suggested the potential for renal protection effect with endogenous EPO administration.
